# (*R*,*S*)-3-Carb­oxy-2-(isoquinolinium-2-yl)propanoate monohydrate

**DOI:** 10.1107/S1600536810018428

**Published:** 2010-05-22

**Authors:** Vladimir Stilinović, Leo Frkanec, Branko Kaitner

**Affiliations:** a Laboratory of General and Inorganic Chemistry, Department of Chemistry, Faculty of Science, University of Zagreb, Horvatovac 102 A, HR-10000 Zagreb, Croatia; b Department of Organic Chemistry and Biochemistry, Ruder Bošković Institute, PO Box 180, HR-10002 Zagreb, Croatia

## Abstract

The title compound, C_13_H_11_NO_4_·H_2_O, is a monohydrate of a betaine exhibiting a positively charged *N*-substituted isoquino­line group and a deprotonated carboxyl group. In the crystal, mol­ecules are connected *via* short O—H⋯O hydrogen bonds between protonated and deprotonated carboxyl groups into chains of either *R* or *S *enanti­omers along [001]. These chains are additionally connected by hydrogen bonding between water mol­ecules and the deprotonated carb­oxy groups of neighbouring mol­ecules.

## Related literature

For the structure of a co-crystal of a quinoline derivative betaine, see: Szafran *et al.* (2002 ▶) and for the structure of a 4-dithio­carboxyl­isoquinoline betaine, see: Matthews *et al.* (1973 ▶). For possible applications of isoquinoline derivatives, see: Katritsky & Pozharskii (2000 ▶). For the preparation of the title compound, see: Flett & Gardner (1952 ▶).
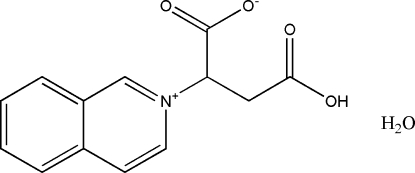


## Experimental

### 

#### Crystal data

C_13_H_11_NO_4_·H_2_O
*M* *_r_* = 263.24
Monoclinic, 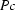

*a* = 10.1030 (15) Å
*b* = 8.0706 (8) Å
*c* = 7.8911 (10) Å
β = 104.282 (14)°
*V* = 623.53 (14) Å^3^
*Z* = 2
Mo *K*α radiation
μ = 0.11 mm^−1^
*T* = 295 K
0.43 × 0.19 × 0.17 mm

#### Data collection

Oxford Diffraction Xcalibur CCD diffractometer
7142 measured reflections
1659 independent reflections
994 reflections with *I* > 2σ(*I*)
*R*_int_ = 0.054

#### Refinement

*R*[*F* ^2^ > 2σ(*F* ^2^)] = 0.076
*wR*(*F* ^2^) = 0.222
*S* = 1.02
1659 reflections
178 parameters
6 restraints
H atoms treated by a mixture of independent and constrained refinement
Δρ_max_ = 0.38 e Å^−3^
Δρ_min_ = −0.23 e Å^−3^

### 

Data collection: *CrysAlis CCD* (Oxford Diffraction, 2006 ▶); cell refinement: *CrysAlis RED* (Oxford Diffraction, 2006 ▶); data reduction: *CrysAlis RED*; program(s) used to solve structure: *SHELXS97* (Sheldrick, 2008 ▶); program(s) used to refine structure: *SHELXL97* (Sheldrick, 2008 ▶); molecular graphics: *ORTEP-3* (Farrugia, 1997 ▶) and *Mercury* (Macrae *et al.*, 2006 ▶); software used to prepare material for publication: *WinGX* (Farrugia, 1999 ▶), *PLATON* (Spek, 2009 ▶) and *PARST* (Nardelli, 1995 ▶).

## Supplementary Material

Crystal structure: contains datablocks global, I. DOI: 10.1107/S1600536810018428/im2195sup1.cif

Structure factors: contains datablocks I. DOI: 10.1107/S1600536810018428/im2195Isup2.hkl

Additional supplementary materials:  crystallographic information; 3D view; checkCIF report

## Figures and Tables

**Table 1:** Hydrogen-bond geometry (Å, °)

*D*—H⋯*A*	*D*—H	H⋯*A*	*D*⋯*A*	*D*—H⋯*A*
O5—H1⋯O2	0.86 (6)	2.05 (6)	2.851 (7)	156 (6)
O5—H2⋯O2^i^	0.86 (6)	2.08 (7)	2.874 (7)	153 (6)
O4—H4⋯O1^ii^	0.82	1.70	2.518 (7)	172

Symmetry codes: (i) 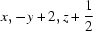; (ii) 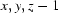.

**Fig. 1.**
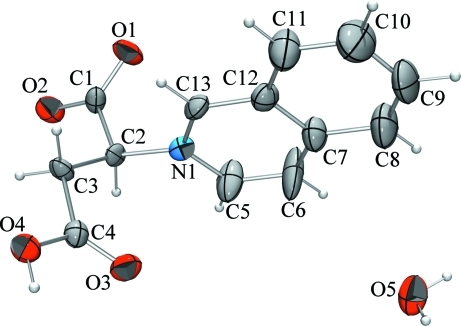
View of (I) with the atom labeling scheme. Displacement ellipsoids of are shown at 30% probability. Hydrogen atoms are shown as spheres of arbitrary radii.

**Fig. 2.**
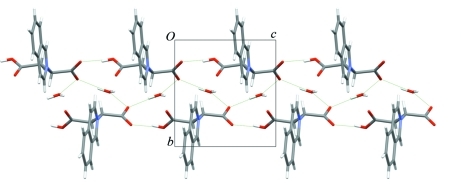
Crystal packing of (I) viewed along the x axis.

## supplementary crystallographic information

### Comment

Isoquinoline derivatives are of interest in synthesizing new fungicides, insecticides, textile assistants, corrosion inhibitors, dye stabilizers, and pharmaceuticals (Katritsky & Pozharskii, 2000) The molecular structure of I is given in Figure 1. The molecule of 3-carboxy-2-isoquinolinium-2-ylpropanoate is a betaine, i.e. a zwitterion containing a quaternary nitrogen atom and a deprotonated carboxyl group. It is the first betaine derived from isoquinoline to be structurally characterised, the only two similar compounds being a quinoline derivative (Szafran *et al.*, 2002) and a 4-dithiocarboxylisoquinoline derivative (Matthews *et al.*, 1973)

The compound crystallises in the space group P*c* with two formula units per unit cell. Molecules of 3-carboxy-2-isoquinolinium-2-ylpropanoate are connected *via* strong hydrogen bonds between protonated and deprotonated carboxyl groups (O4—H4···O1 2.518 (7) Å, (*x*, *y*, -1+*z*)) along the *c* axis. Water molecules bridge two deprotonated carboxyl groups of neighbouring molecules along chains (O5—H2···O2 2.874 (7) Å, (*x*, 2- *y*, 1/2 + *z*) and O5—H1···O2 2.851 (7) Å). Chains consist of either *R* or *S *enantiomers and each chain is interconnected by water molecules to a neighbouring chain in which the molecules are of opposite chirality, thus forming double chains about the glide plane.

### Experimental

The title compound (I) was prepared according to a method described earlier (Flett & Gardner, 1952). Separate solutions are prepared of isoquinoline (1.17 ml; 10 mmol) and maleic acid (1.16 g; 10 mmol) in anhydrous ether. Upon mixing, isoquinolinium maleate precipitates. This precipitate is separated by filtration, washed, and dried. It is then rapidly heated to its melting point at 103 °C and held at this temperature for a few minutes. Rapid conversion to the betaine takes place. The betaine is then purified by dissolving it in hot water and treatment with animal charcoal. The solution was set aside for the formation of crystals, yield is 79 %. Crystals suitable for crystallographic study were grown from a solution of (I) in water by slow evaporation at room temperature.

### Refinement

The hydrogen atoms of the water molecule were located in the difference Fourier map and refined isotropically with the O–H distance restrained to 0.857 (2) Å. All other H atoms were placed geometrically and included in the refinement in the riding-model approximation with *U*_iso_ = 1.2 *U*_eq_ for hydrogen atoms bonded to carbon and *U*_iso_ = 1.5 *U*_eq_ for the hydroxyl hydrogen. To the quinolinium subunit rigid bond restraints were applied. Since there are no heavy atoms in the structure the Flack parameter was meaningless due to a large s.u., and the Friedel pairs were merged for the final refinement.

### Crystal data

**Table:**

C_13_H_11_NO_4_·H_2_O	*F*(000) = 276
*M**_r_* = 263.24	*D*_x_ = 1.402 Mg m^−^^3^
Monoclinic, *P**c*	Mo *K*α radiation, λ = 0.71073 Å
Hall symbol: P -2yc	Cell parameters from 275 reflections
*a* = 10.1030 (15) Å	θ = 4.6–52.0°
*b* = 8.0706 (8) Å	µ = 0.11 mm^−^^1^
*c* = 7.8911 (10) Å	*T* = 295 K
β = 104.282 (14)°	Prism, colourless
*V* = 623.53 (14) Å^3^	0.43 × 0.19 × 0.17 mm
*Z* = 2	

### Data collection

**Table:**

Oxford Diffraction Xcalibur CCD diffractometer	994 reflections with *I* > 2σ(*I*)
Radiation source: fine-focus sealed tube	*R*_int_ = 0.054
graphite	θ_max_ = 29°, θ_min_ = 3.9°
ω scan	*h* = −13→13
7142 measured reflections	*k* = −11→11
1659 independent reflections	*l* = −10→10

### Refinement

**Table:**

Refinement on *F*^2^	Primary atom site location: structure-invariant direct methods
Least-squares matrix: full	Secondary atom site location: difference Fourier map
*R*[*F*^2^ > 2σ(*F*^2^)] = 0.076	Hydrogen site location: inferred from neighbouring sites
*wR*(*F*^2^) = 0.222	H atoms treated by a mixture of independent and constrained refinement
*S* = 1.02	*w* = 1/[σ^2^(*F*_o_^2^) + (0.1361*P*)^2^] where *P* = (*F*_o_^2^ + 2*F*_c_^2^)/3
1659 reflections	(Δ/σ)_max_ < 0.001
178 parameters	Δρ_max_ = 0.38 e Å^−^^3^
6 restraints	Δρ_min_ = −0.23 e Å^−^^3^

### Special details

**Table:**

Geometry. All esds (except the esd in the dihedral angle between two l.s. planes) are estimated using the full covariance matrix. The cell esds are taken into account individually in the estimation of esds in distances, angles and torsion angles; correlations between esds in cell parameters are only used when they are defined by crystal symmetry. An approximate (isotropic) treatment of cell esds is used for estimating esds involving l.s. planes.
Refinement. Refinement of *F*^2^ against ALL reflections. The weighted *R*-factor wR and goodness of fit *S* are based on *F*^2^, conventional *R*-factors *R* are based on *F*, with *F* set to zero for negative *F*^2^. The threshold expression of *F*^2^ > σ(*F*^2^) is used only for calculating *R*-factors(gt) etc. and is not relevant to the choice of reflections for refinement. *R*-factors based on *F*^2^ are statistically about twice as large as those based on *F*, and *R*- factors based on ALL data will be even larger.

### Fractional atomic coordinates and isotropic or equivalent isotropic displacement parameters (Å^2^)

**Table:**

	*x*	*y*	*z*	*U*_iso_*/*U*_eq_	
O5	0.9214 (4)	0.9884 (8)	0.9683 (6)	0.0817 (17)	
H1	0.865 (6)	0.928 (9)	0.895 (8)	0.085*	
H2	0.885 (7)	1.014 (11)	1.052 (7)	0.086*	
O3	0.5000 (5)	0.8301 (5)	0.0404 (6)	0.0581 (12)	
O1	0.5719 (5)	0.7025 (7)	0.6870 (6)	0.0664 (14)	
O4	0.6768 (5)	0.6788 (6)	0.0104 (6)	0.0576 (12)	
H4	0.6439	0.6955	−0.094	0.086*	
C2	0.5675 (6)	0.8088 (7)	0.4031 (6)	0.0340 (11)	
H2A	0.5661	0.9273	0.3756	0.041*	
N1	0.4242 (5)	0.7538 (5)	0.3583 (6)	0.0375 (10)	
C4	0.6005 (6)	0.7514 (6)	0.1025 (7)	0.0372 (12)	
C1	0.6317 (6)	0.7933 (7)	0.6011 (7)	0.0384 (12)	
O2	0.7403 (5)	0.8672 (6)	0.6568 (6)	0.0584 (12)	
C12	0.2584 (7)	0.5400 (7)	0.3023 (7)	0.0448 (13)	
C3	0.6546 (6)	0.7251 (7)	0.2928 (7)	0.0415 (13)	
H3A	0.7469	0.7684	0.3277	0.05*	
H3B	0.6589	0.6071	0.3167	0.05*	
C13	0.3918 (7)	0.5934 (7)	0.3442 (8)	0.0446 (13)	
H13	0.4616	0.5153	0.3632	0.054*	
C7	0.1528 (8)	0.6561 (9)	0.2716 (11)	0.0632 (18)	
C6	0.1918 (8)	0.8250 (10)	0.287 (2)	0.115 (5)	
H6	0.1246	0.9064	0.2687	0.137*	
C10	0.0902 (11)	0.3265 (12)	0.251 (2)	0.121 (5)	
H10	0.0679	0.2145	0.2447	0.146*	
C8	0.0157 (8)	0.6063 (11)	0.2240 (15)	0.088 (3)	
H8	−0.0545	0.6839	0.1992	0.105*	
C11	0.2251 (9)	0.3727 (10)	0.2954 (16)	0.093 (3)	
H11	0.2935	0.2928	0.3209	0.112*	
C9	−0.0115 (9)	0.4417 (12)	0.2152 (14)	0.089 (3)	
H9	−0.102	0.4065	0.184	0.107*	
C5	0.3220 (8)	0.8692 (9)	0.3285 (14)	0.083 (3)	
H5	0.3444	0.9812	0.3373	0.099*	

### Atomic displacement parameters (Å^2^)

**Table:**

	*U*^11^	*U*^22^	*U*^33^	*U*^12^	*U*^13^	*U*^23^
O5	0.058 (3)	0.088 (4)	0.093 (4)	0.004 (3)	0.009 (3)	−0.029 (3)
O3	0.070 (3)	0.058 (3)	0.044 (2)	0.011 (3)	0.010 (2)	0.012 (2)
O1	0.087 (4)	0.088 (3)	0.0270 (19)	−0.019 (3)	0.020 (2)	0.006 (2)
O4	0.074 (3)	0.068 (3)	0.032 (2)	0.004 (2)	0.0153 (19)	−0.004 (2)
C2	0.050 (3)	0.035 (3)	0.0162 (18)	−0.003 (2)	0.0079 (19)	0.0013 (18)
N1	0.049 (3)	0.030 (2)	0.031 (2)	0.0015 (19)	0.0078 (18)	−0.0013 (17)
C4	0.045 (3)	0.036 (3)	0.030 (3)	−0.011 (2)	0.008 (2)	−0.013 (2)
C1	0.040 (3)	0.042 (3)	0.034 (3)	0.000 (2)	0.009 (2)	0.002 (2)
O2	0.056 (3)	0.077 (3)	0.037 (2)	−0.020 (2)	0.0025 (18)	0.002 (2)
C12	0.045 (3)	0.047 (3)	0.039 (3)	−0.004 (3)	0.003 (2)	0.004 (2)
C3	0.048 (3)	0.043 (3)	0.029 (3)	0.000 (3)	0.002 (2)	0.007 (2)
C13	0.049 (4)	0.035 (3)	0.048 (3)	0.002 (3)	0.007 (3)	0.003 (2)
C7	0.043 (4)	0.054 (4)	0.090 (5)	0.003 (3)	0.013 (3)	−0.012 (3)
C6	0.037 (5)	0.043 (4)	0.244 (15)	0.004 (3)	−0.002 (6)	−0.030 (6)
C10	0.066 (6)	0.059 (5)	0.215 (15)	−0.022 (4)	−0.010 (7)	0.032 (7)
C8	0.039 (4)	0.073 (5)	0.142 (8)	−0.008 (4)	0.006 (4)	−0.010 (6)
C11	0.067 (6)	0.042 (4)	0.152 (9)	−0.010 (4)	−0.008 (6)	−0.001 (5)
C9	0.047 (5)	0.085 (6)	0.126 (8)	−0.029 (4)	0.003 (4)	−0.006 (5)
C5	0.045 (4)	0.034 (3)	0.157 (8)	0.008 (3)	0.004 (4)	−0.024 (4)

### Geometric parameters (Å, °)

**Table:**

O5—H1	0.86 (6)	C12—C7	1.396 (9)
O5—H2	0.86 (6)	C3—H3A	0.97
O3—C4	1.195 (7)	C3—H3B	0.97
O1—C1	1.250 (7)	C13—H13	0.93
O4—C4	1.320 (7)	C7—C8	1.402 (11)
O4—H4	0.82	C7—C6	1.416 (11)
C2—N1	1.471 (7)	C6—C5	1.324 (11)
C2—C3	1.538 (7)	C6—H6	0.93
C2—C1	1.543 (6)	C10—C9	1.364 (13)
C2—H2A	0.98	C10—C11	1.372 (12)
N1—C13	1.333 (7)	C10—H10	0.93
N1—C5	1.368 (8)	C8—C9	1.354 (12)
C4—C3	1.481 (7)	C8—H8	0.93
C1—O2	1.231 (7)	C11—H11	0.93
C12—C13	1.375 (8)	C9—H9	0.93
C12—C11	1.389 (10)	C5—H5	0.93
			
H1—O5—H2	109 (7)	H3A—C3—H3B	107.8
C4—O4—H4	109.5	N1—C13—C12	122.0 (5)
N1—C2—C3	113.5 (4)	N1—C13—H13	119
N1—C2—C1	111.2 (4)	C12—C13—H13	119
C3—C2—C1	112.4 (4)	C12—C7—C8	121.1 (7)
N1—C2—H2A	106.4	C12—C7—C6	116.5 (7)
C3—C2—H2A	106.4	C8—C7—C6	122.3 (7)
C1—C2—H2A	106.4	C5—C6—C7	121.3 (7)
C13—N1—C5	119.2 (6)	C5—C6—H6	119.3
C13—N1—C2	121.3 (5)	C7—C6—H6	119.3
C5—N1—C2	119.5 (5)	C9—C10—C11	121.2 (8)
O3—C4—O4	124.2 (5)	C9—C10—H10	119.4
O3—C4—C3	123.8 (5)	C11—C10—H10	119.4
O4—C4—C3	112.0 (5)	C9—C8—C7	118.0 (8)
O2—C1—O1	126.7 (5)	C9—C8—H8	121
O2—C1—C2	116.0 (5)	C7—C8—H8	121
O1—C1—C2	117.2 (5)	C10—C11—C12	119.3 (8)
C13—C12—C11	121.9 (6)	C10—C11—H11	120.3
C13—C12—C7	119.5 (6)	C12—C11—H11	120.3
C11—C12—C7	118.6 (7)	C8—C9—C10	121.7 (8)
C4—C3—C2	113.0 (4)	C8—C9—H9	119.2
C4—C3—H3A	109	C10—C9—H9	119.2
C2—C3—H3A	109	C6—C5—N1	121.4 (7)
C4—C3—H3B	109	C6—C5—H5	119.3
C2—C3—H3B	109	N1—C5—H5	119.3

### Hydrogen-bond geometry (Å, °)

**Table:**

*D*—H···*A*	*D*—H	H···*A*	*D*···*A*	*D*—H···*A*
O5—H1···O2	0.86 (6)	2.05 (6)	2.851 (7)	156 (6)
O5—H2···O2^i^	0.86 (6)	2.08 (7)	2.874 (7)	153 (6)
O4—H4···O1^ii^	0.82	1.70	2.518 (7)	172

Symmetry codes: (i) *x*, −*y*+2, *z*+1/2; (ii) *x*, *y*, *z*−1.
